# Recent Progress in Devices Based on Magnetoelectric Composite Thin Films

**DOI:** 10.3390/s21238012

**Published:** 2021-11-30

**Authors:** Deepak Rajaram Patil, Ajeet Kumar, Jungho Ryu

**Affiliations:** School of Materials Science and Engineering, Yeungnam University, Gyeongsan 38541, Korea; deepphy24@gmail.com (D.R.P.); jkajeet@yahoo.co.in (A.K.)

**Keywords:** magnetoelectric composites, thin film, piezoelectric effect magnetostriction

## Abstract

The strain-driven interfacial coupling between the ferromagnetic and ferroelectric constituents of magnetoelectric (ME) composites makes them potential candidates for novel multifunctional devices. ME composites in the form of thin-film heterostructures show promising applications in miniaturized ME devices. This article reports the recent advancement in ME thin-film devices, such as highly sensitive magnetic field sensors, ME antennas, integrated tunable ME inductors, and ME band-pass filters, is discussed. (Pb_1__−x_Zr_x_)TiO_3_ (PZT), Pb(Mg_1/3_Nb_2/3_)O_3_-PbTiO_3_ (PMN-PT), Aluminium nitride (AlN), and Al_1__−x_Sc_x_N are the most commonly used piezoelectric constituents, whereas FeGa, FeGaB, FeCo, FeCoB, and Metglas (FeCoSiB alloy) are the most commonly used magnetostrictive constituents in the thin film ME devices. The ME field sensors offer a limit of detection in the fT/Hz^1/2^ range at the mechanical resonance frequency. However, below resonance, different frequency conversion techniques with AC magnetic or electric fields or the delta-E effect are used. Noise floors of 1–100 pT/Hz^1/2^ at 1 Hz were obtained. Acoustically actuated nanomechanical ME antennas operating at a very-high frequency as well as ultra-high frequency (0.1–3 GHz) range, were introduced. The ME antennas were successfully miniaturized by a few orders smaller in size compared to the state-of-the-art conventional antennas. The designed antennas exhibit potential application in biomedical devices and wearable antennas. Integrated tunable inductors and band-pass filters tuned by electric and magnetic field with a wide operating frequency range are also discussed along with miniaturized ME energy harvesters.

## 1. Introduction

Magnetoelectric (ME) composites composed of two distinct magnetostrictive and piezoelectric constituents have shown great promise as energy harvesters, gyrators, magnetic field sensors, and transducers, [1,2,3,4,5] owing to their multifunctional properties, which are absent in the individual constituents [1,2]. ME composites are derived by the ME effect—a combination of the magnetostriction in the magnetostrictive constituent and piezoelectric effect in the piezoelectric phase [2]. ME effects are distinguished as the direct ME effect (magnetic field, H, control of electric polarization, P) and converse ME effect (electric field, E, control of magnetization, M). The mechanism of both the ME effects in ME composites are derived as follows [6]:

Direct ME effect (DME):


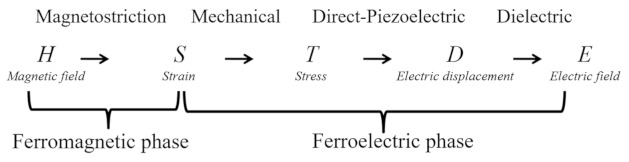


Converse ME effect (CME):


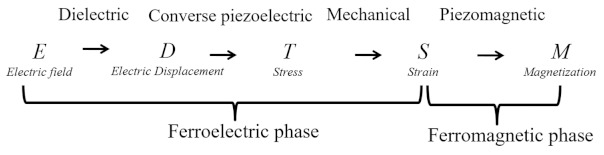


In both cases, the ME effect is a result of the strain-mediated interface coupling between the piezoelectric and magnetostrictive phases. In DME, an external magnetic field induces magnetostrictive strain in the magnetostrictive phase through the magnetostriction effect, which acts as a stress to the piezoelectric phase, resulting in electric polarization through the direct piezoelectric effect. In CME, the applied electric field generates stress in the piezoelectric layer via converse piezoelectric effect, which induces strain in the magnetostrictive phase, resulting in magnetization through the piezomagnetic effect. 

The magnetic field induced ME coupling in the composite can be represented as [1]:

(1) $$\alpha_{ij}^{H}=\left(\frac{\partial P_{i}}{\partial H_{j}}\right)=\varepsilon_{0}\varepsilon_{r}\left(\frac{\partial E_{i}}{\partial H_{j}}\right)=\frac{\varepsilon_{0}\varepsilon_{r}\left(\frac{\partial V}{\partial H}\right)}{t}=\varepsilon_{0}\varepsilon_{r}\alpha_{E}$$

where α is the ME coefficient, *E* = (*V*/*t*), *V* is the induced voltage, *t* is the thickness, and $\alpha_{E}$ is the ME voltage coefficient. $\alpha \;\mathrm{and}\;\alpha_{E}$ are expressed in s/m and V/cm·Oe, respectively. In general, $\alpha_{E}=kdq$, where *k* is the interfacial coupling factor, *d* is the piezoelectric coefficient of the piezoelectric phase, *q* is the piezomagnetic coefficient, and *q* = d*λ*/d*H* (λ) is the magnetostriction of the magnetostrictive phase.

**Figure 1:**
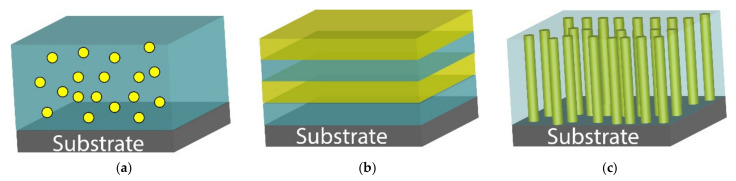
Schematic of (**a**) 0–3, (**b**) 2–2, and (**c**) 1–3 type magnetoelectric composite nanostructures.

Different types of ME composites fabricated as bulk, thick film, and thin film have been extensively investigated, according to the device application. In particular, bulk ME composites and ME thick films showed very large room temperature ME coupling. These composites are operated in different configurations such as L-T, T-T, and L-L (L represents longitudinal and T represents transverse), depending on the directions *H* and *P*. The highest $\alpha_{E}$ of 20 V/cm·Oe was reported for bulk ME laminates at off-resonance frequencies (*f* = 1–1000 Hz). The $\alpha_{E}$ of ME composites is enhanced by 10^2^ orders when operated at the electromechanical resonance, *f*_r_. The maximum $\alpha_{E}\;$ of >1000 V/cm·Oe was reported at *f*_r_. The large enhancement under resonance conditions can be utilized in effectively transducing magnetic energy into electric energy. Although these composites possess ideal ME coupling, they still exhibit inherent limitations, such as imperfect interface coupling, bulk size, and sintering during synthesis. However, recent developments in thin-film fabrication techniques have provided more alternatives for the fabrication of different ME heterostructures, where ferromagnetic and ferroelectric constituents can be coupled at an atomic level to achieve good interfacial coupling in strain-mediated ME composites [4]. In this context, different types of ME thin films, particulate heterostructures (0–3), vertical heterostructures (1–3), and laminate (2–2) (Figure 1) have been fabricated using pulsed laser deposition (PLD) [7,8], molecular beam epitaxy (MBE) [9,10], metal-organic chemical vapor deposition (MOCVD) [11], spin coating [12], and sputtering [13,14]. In particular, epitaxial growth techniques (PLD, MBE, and sputtering) are more feasible than the rest for fabricating single crystalline or self-assembled oxide heterostructures with more control over the structures. In comparison with bulk materials, thin-film ME composites have some distinctive advantages, such as low interface losses, complementary metal–oxide–semiconductor (CMOS)-compatible fabrication process, light weight, and high spatial resolution. Therefore, they are more promising candidates for integrated RF/microwave ME devices. In this review, we focus on current developments in different ME thin film devices, such as nanoelectromechanical systems (NEMS) ME resonators, integrated magnetic tunable inductors, tunable RF bandpass filters, novel NEMS ME antennas, and miniaturized energy harvesters. 

## 2. Thin-Film Magnetic Sensors

ME composites are suitable as high-sensitivity magnetic field sensors because of their high voltage coefficient. They are an alternative to the presently available stat-of-the-art magnetic sensors based on the SQUID, Hall effect, flux gate, etc., for possible applications in bio-magnetic field detection such as magnetocardiography (MCG) [15], magnetomyography (MMG) [16], and magnetic particle imaging [17]. These sensors have the advantages of room-temperature operation and low power consumption. The selection of the constituent phases is important for realizing good ME sensor performance. The performance of the ME sensor was evaluated based on three parameters: sensitivity, noise, and signal-to-noise ratio (SNR). The best SNR was observed for piezoelectric constituents with large $\frac{d_{31}}{{\left(\varepsilon_{0\varepsilon_{33}tan\delta }\right)}^{1/2}}$ [18]. Some of the most commonly used piezoelectric materials are (Pb_1−x_Zr_x_)TiO_3_ (PZT) or PMN-xPT for bulk ME sensors and PZT, AlScN, and AlN for thin-film ME sensors [19,20,21,22,23]. Similarly, quartz, LiNbO_3_, and LiTaO_3_ were used as piezoelectric single crystal substrates for ME sensors based on surface acoustic waves (SAWs). Soft magnetic alloys, such as Terfenol-D (Tb_x_Dy_1__−x_Fe_2_), FeGa, FeGaB, FeCo, FeCoB, and Metglas (FeCoSiB alloy), with high piezomagnetic coefficients and permeabilities, have been utilized as magnetostrictive constituents [19]. ME sensors are characterized by the limit of detection (LOD) and the ratio of ME voltage to noise voltage density. In other words, LOD is the minimum detectable ac magnetic field, at which the ME voltage reaches the noise floor. In LOD measurement, the amplitude of ac magnetic field is continuously decreased starting from the values which are about three orders higher than the minimum detectable magnetic field. The LOD was determined by linearly fitting the output voltage to the noise floor. The intersection of the two lines gives the LOD at which the SNR becomes 1. The LOD is expressed in units of T/Hz^1/2^ [18].

ME sensors based on bulk ME composites were developed earlier. The magnetic field detectable by ME bulk sensors is in the range of 1–10 pT at *f* = 1 Hz, which is sufficient for DC and geomagnetic signal detection. However, ME bulk sensors are bulky and difficult to miniaturize for spatially resolved measurements of sensor arrays. In contrast, ME thin-film sensors allow for size reduction for spatially resolved measurements. They possess perfect interfacial mechanical coupling for effective strain transfer between the two phases and large ME coupling due to high quality factors at the resonance frequency. The vacuum sealing can further enhance the quality factor of the sensors. Moreover, the resonance frequency of thin-film sensors can be tuned in the desired range by designing cantilever structures. 

Most thin-film ME sensors are fabricated by micromachining Si cantilevers with typical dimensions of approximately 25 mm (length) × 2.2 mm (width) × 300 µm (thickness). The ME composite constituent phases (thickness of a few nanometers) are deposited on the top and rear sides of the double-sized polished Si substrate. ME sensors are classified into three categories based on their sensitivity and operational bandwidth [24,25]. The first type of ME sensor is based on direct measurement of the ME voltage coefficient of ME composites, where sensor sensitivity is derived from the output ME voltage and related to the DC magnetic field and operational frequency. The second type is based on electric and magnetic frequency conversion techniques, where the nonlinearity of magnetostriction is used. The third type of sensor is based on the delta-E effect. The delta *E* effect creates changes in the Young’s modulus of the magnetostrictive phase under an applied magnetic field [26,27]. 

### 2.1. ME Cantilever Sensors

**Figure 2:**
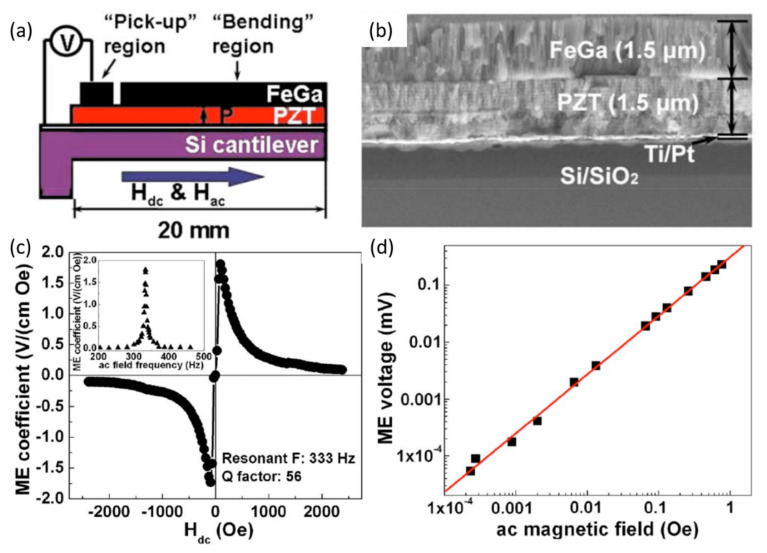
(**a**) Schematic representation of the fabricated FeGa/PZT ME sensor. (**b**) SEM image of FeGa/PZT ME sensor. (**c**) *H*_dc_ dependent of $\alpha_{E}$ of a FeGa/PZT sensor at its resonant frequency. Inset: Frequency‒dependent $\alpha_{E}$. (**d**) $\alpha_{E}$ as a function of magnitude of an AC field at 333 Hz under a DC magnetic field of 90 Oe. Reprinted with permission from ref. [24]. Copyright 2009 AIP Publishing.

The ME voltage coefficient-based ME sensors are based on the resonant cantilever design, which uses enhanced ME coupling at mechanical resonance under AC magnetic field (*H*_ac_). $\alpha_{E}$ is measured as a function of the frequency of *H*_ac_ under a constant DC magnetic field (*H*_dc_). Here, both *H*_ac_ and *H*_dc_ were applied along the length of the cantilever beam. The ME sensor shows two to three orders of enhancement at the resonance frequency (*f*_res_). Zhao et al. [24] reported a thin film-based ME sensor composed of PZT film (sol-gel-derived) and Fe_0.7_Ga_0.3_ film (sputter deposited) on an Si cantilever. A schematic of the fabricated PZT/ Fe_0.7_Ga_0.3_ ME sensor is shown in Figure 2a, and its cross-sectional scanning electron microscopy (SEM) image is shown in Figure 2b. A PZT (1.5 µm)/Fe_0.7_Ga_0.3_ (1.5 µm) heterostructure was deposited on a 4.5 mm × 20 mm × 35 µm Si beam. The SEM image clearly shows the interface boundary between the PZT and FeGa columnar microstructures. Figure 2c exhibits *H*_dc_ dependent $\alpha_{E}$ of the PZT/Pt/FeGa film at a resonance frequency of 333 Hz. The inset of Figure 2c shows the frequency dependence of $\alpha_{E}$ of the PZT/Pt/FeGa film, where resonance frequency of 333 Hz was observed. A maximum $\alpha_{E}$ of 1.81 V/cm·Oe was obtained at an *H*_dc_ of 90 Oe. To evaluate the AC magnetic field sensitivity, AC magnetic field dependent ME voltage was measured as shown in Figure 2d. To improve the sensitivity of the device, two pick up regions with area of 1 mm^2^ were connected in series. The PZT/Pt/FeGa ME sensor was able to detect AC field of 2.3 × 10^−8^ T with a noise floor of 50 nV. Piorra et al. [17] reported an improvement in the LOD of an ME sensor by replacing PZT with an AlN layer. They obtained a four-times higher LOD of 2.6 pT/Hz^1/2^ than an ME sensor with PZT. 

**Figure 3:**
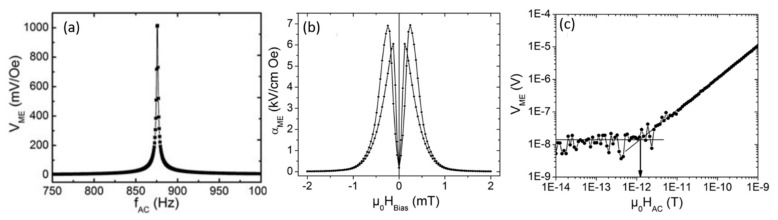
ME study of a FeCoSiB/AlN ME sensor. (**a**) ME voltage at electromechanical resonance (*f*_r_ = 876 Hz). (**b**) ME voltage coefficient versus DC magnetic field at *f*_r_. (**c**) Output voltage versus AC magnetic field at *f*_r_ with optimum *H*_dc_. Reprinted with permission from ref. [28]. Copyright 2015 AIP Publishing.

Robisch et al. [28] fabricated an ME sensor composed of a Ta/FeCoSiB/Ta/Pt/AlN/Cr/Au (FeCoSiB/AlN) ME composite thin film on an Si cantilever. Figure 3a shows the frequency-dependent ME voltage (*V*_ME_) of the. The highest *V*_ME_ of 1 V/Oe was obtained at a resonance frequency, *f*_r_, of 876 Hz. Figure 3b shows $\alpha_{E}$ as a function of *H*_dc_ at *f*_r_ = 876 Hz for FeCoSiB/AlN ME sensor. The maximum $\alpha_{E}$ of 6.9 kV/cm·Oe was observed at an optimum *H*_dc_ of ± 2.4 Oe. The limit of detection (LOD) was determined at the optimum *H*_dc_ (2.4 Oe) and *H*_ac_ (867 Hz). Figure 3c shows the variation of output voltage as a function of *H*_ac_. With decreasing *H*_ac_, *V*_ME_ initially decreases linearly by several orders in magnitude of *H*_ac_ and then suppressed by the noise floor at lower *H*_ac_, indicating independency on *H*_ac_. The LOD was determined by linearly fitting the output voltage to the noise floor. The intersection of the two lines (indicated by the arrow in Figure 3c) gives the LOD at which the SNR becomes 1. The LOD is found to be in the order of 1 pT/Hz^1/2^. Yarar et al. [29] fabricated an inverse bilayer ME sensor composed of an Si/SiO_2_/Pt/FeCoSiB/AlN layer stack, where FeCoSiB was deposited first on the Si substrate followed by AlN on it, without heating the substrate to preserve the amorphous nature of FeCoSiB. Transmission electron microscopy analysis confirmed the epitaxial growth of AlN along c-axis originating from the Pt-AlN interface. As a result, an LOD of approximately 400 fT/Hz^1/2^ was obtained at a resonance frequency of 867 Hz with a high $\alpha_{E}$ of 5 kV/cm·Oe. However, the sensitivity of these ME sensors is restricted by environmental noise sources such as vibrations and acoustic waves, which lead to the thermomechanical noise in the cantilever. 

**Figure 4:**
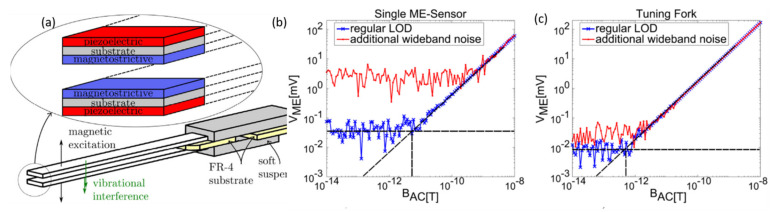
(**a**) Tuning fork structure consisting of two identical cantilever ME sensors placed on top and bottom of the mounting block. LOD plots of (**b**) a single ME sensor and (**c**) a tuning fork ME sensor with and without additional wideband noise. Reprinted with permission from ref. [30]. Copyright 2021 Elsevier Publishing.

Salzer et al. [30] introduced a new strategy of utilizing tuning fork-like structures to improve the LOD by reducing the noise caused by vibrations and acoustic waves. Figure 4a shows the tuning fork structure consisting of two identical asymmetric cantilever ME sensors placed on the top and bottom of the mounting block. The two sensors were placed opposite to each other, to get the two different signals from the vibration and magnetic excitations. Under a magnetic field, two sensors deflect opposite to each other, as shown by the black arrows in Figure 4a, whereas both cantilevers deflect in the same direction (green arrows) under vibrational interference. This configuration led to distinguishable contributions from the magnetic field and vibration interference. The ME sensors consisted of Ta (5 nm)/FeCoSiB (4 µm)/Si (300 µm)/ZrO_2_ (300 nm)/PZT 2 (µm)/Cr (5 nm)/Au (100 nm). The LOD measurements with one sensor and fork-like structure (two sensors) are shown in Figure 4b,c, respectively. The wideband noise was superimposed using piezoelectric loudspeakers. Without external noise, the tuning fork exhibited a noise level that is four times lower than that of a single ME sensor (blue line). With superimposed noise, the noise level was increased by two orders of magnitude for the single ME sensor, whereas it is increased by a factor of only four for the fork structure. The LOD was found to be 500 fT/(Hz)^1/2^, which is much higher than the LOD of single ME sensor. 

Most of the ME sensors mentioned above are based on AlN as the piezoelectric constituent of the ME sensor. Recently, Su et al. [31] replaced AlN thin films in an ME sensor with AlScN to improve the piezoelectric coefficient of AlN. The alloying of AlN with Sc resulted in the increasing piezoelectric coefficient ($\alpha_{E}$) by 2 (1.4) times in turns increase in the LOD of by a factor of 1.85 for an AlScN-based ME sensor. 

### 2.2. Self-Biased ME Sensors

Most of the above-mentioned ME sensors require an optimized *H*_dc_ (Figure 2c) along with *H*_ac_ to enhance the ME sensitivity. Therefore, an extra *H*_dc_ source in the ME sensor assembly is inevitable, which can result in extra space, cross-talk with sensors, and the addition of magnetic noise. Therefore, to overcome these limitations, Lage et al. [32] introduced the concept of exchange bias of a magnetostrictive constituent. The exchange biasing is generated by shifting of exchange bias field (*H*_EB_) of the magnetization-magnetic field curve through ferromagnetic—antiferromagnetic exchange coupling. Lage et al. fabricated two different ME composite thin films consisting of AlN and multilayers of Ta/Cu/Mn_70_Ir_30_/Fe_70.2_Co_7.8_Si_12_B_10_ or Ta/Cu/Mn_70_Ir_30_/Fe_50_Co_50_. The magnetostriction curve was shifted to obtain the maximum piezomagnetic coefficient at zero *H*_dc_ by optimizing the thickness of the ferromagnetic layers and angle dependency of the exchange bias field. These self-biased ME sensors showed a high magnetic field sensitivity of 10 pT/Hz^1/2^ with a maximum $\alpha_{E}$ of 96 V/cm·Oe at *f*_r_ [32].

### 2.3. Sensitivity Enhancement of an ME Sensor Using the Frequency Conversion Technique

**Figure 5:**
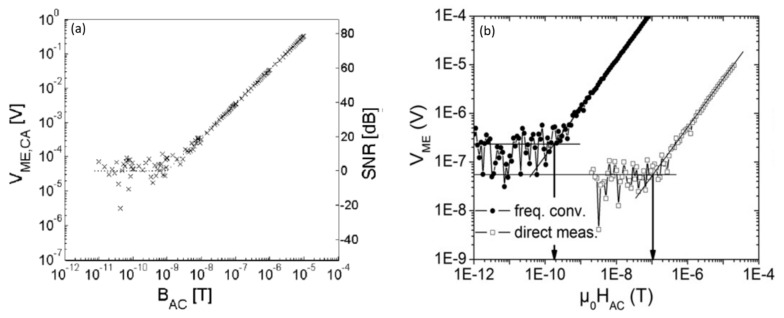
(**a**) Sensitivity of the modulated AlN/FeCoBSi sensor at 1 Hz. (**b**) The direct LOD measurement of AlN/FeCoBSi sensor at *f*_AC_ = 10 Hz and *H*_Bias_ = −5 Oe and by frequency conversion with an RMS amplitude of *H*_mod_ = 3.96 Oe and *f*_mod_ = 1001 Hz. Adapted with permission from ref. [33]. Copyright 2021 Elsevier Publishing.

The above-mentioned ME sensors exhibit high sensitivity in the pT/Hz^1/2^ range at *f*_r_. However, the bandwidth of the resonance frequency is very narrow—a few Hz—and their sensitivity was drastically reduced (few nT/Hz^1/2^) at the low-frequencies (0.1–100 Hz). Many biomedical applications generally require high sensitivity in the low-frequency regime. In the low-frequency regime, the sensitivity of the cantilever-type ME sensors decreases drastically, owing to increasing noise levels and decreasing signal levels. Therefore, the concept of electric or magnetic frequency conversion (EFC or MFC) was introduced to allow wideband operations with high sensitivity at low frequencies [33,34]. The MFC or EFC techniques match the arbitrary frequencies outside the resonance to the resonance frequency of the alternating and time-varying bias field, called the modulation field *H*_mod_. A large amplitude of *H*_mod_ applied to the sensor causes a periodic modulation of the piezomagnetic coefficient. In the presence of an AC magnetic field (*f*_ac_), the frequency of *H*_mod_ (*f*_mod_) is applied such that the arbitrary frequency (*f*_ac_) matches the resonance frequency (*f*_res_), that is, *f*_res_ = *f*_mod_ ± *f*_ac_. By adjusting *f*_mod_, the resonance enhancement of the ME voltage output was achieved at arbitrary frequencies (Figure 5a). Using the MFC technique, Jahns et al. [33] achieved a sensitivity enhancement in an AlN/FeCoBSi ME sensor. Figure 5b shows the LOD measurement for the AlN/FeCoBSi ME sensor with direct measurement and by using the MFC conversion technique. They achieved a sensitivity enhancement of 1000 times using MFC conversion with an RMS amplitude of *H*_mod_ = 3.96 Oe and *f*_mod_ = 1001 Hz. Similarly, Robisch et al. [35] enhanced the LOD by approximately one order of magnitude for an exchange-biased ME sensor based on AlN/FeCoBSi. An LOD of 180 pT/Hz^1/2^ at 10 Hz using the MFC technique was obtained. Furthermore, Robisch et al. obtained the highest LOD of 800 pT/Hz^1/2^ for a 10 Hz signal using the MFC technique. Klug et al. [36] reported the use of antiparallel exchanged biased multilayer ME sensors to improve performance by the restricting the magnetic domain activity. A drastic improvement in the sensor noise was observed, with a reduction in noise level by a few orders of magnitude.

**Figure 6:**
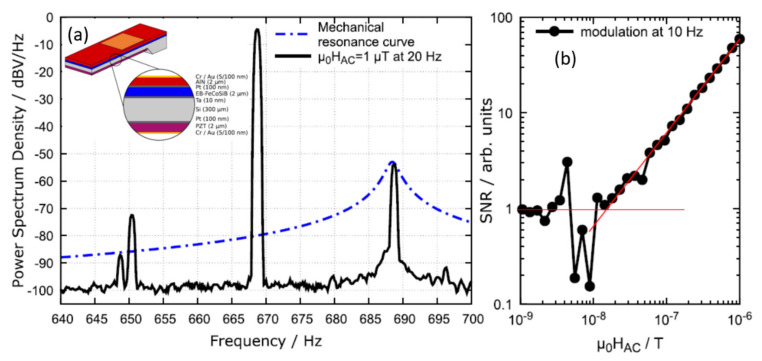
(**a**) Output spectrum of AIN/FeCoSiB/PZT sensor with *f*_mod_ at 669 Hz. A 20 Hz sinusoidal field of 1 µT is applied to the sensor which results in the two sidebands *f*_mod_ ± 20 Hz. Inset shows the fabricated ME sensor consisting of three active layers. (**b**) Linearity measurement at 10 Hz, where an LOD of 10 nT/Hz^1/2^ was obtained. Reprinted with permission from ref. [34]. Copyright 2021 AIP Publishing.

The EFC technique is the same as that of the MFC technique, except that the electric modulation frequency is chosen instead of the magnetic modulation frequency. In EFC, piezoelectric-induced periodic actuation of the ME sensor is utilized. The ME sensor consists of three different functional layers: a nonlinear piezoelectric actuation layer (PZT), an exchange-biased magnetostrictive layer (FeCoSiB), and a sensing layer (AlN), as shown in the inset of Figure 6a. Figure 6a shows the modulation frequency of 669 Hz applied to the PZT layer, which results in sum of the desired magnetic frequency (20 Hz) and modulation frequency (669 Hz) to match the resonant frequency of the ME sensor (689 Hz). Figure 6b shows the LOD measurement at 10 Hz, where an LOD of 10 nT/Hz^1/2^ was achieved. Hayes et al. [34] also obtained an LOD for the AlN/FeCoSiB/PZT ME sensor in the range of nT/Hz^1/2^ at a low frequency of 200 mHz. 

Another method for enhancing the sensitivity at low frequencies is using the delta-E effect [37]. The delta-E effect causes a magnetic field-induced change in the elastic modulus, *E*, of the magnetostrictive materials. Under the applied magnetic field, magnetostrictive strain couples with *E*, which results in the reduction in *E*. Therefore, the magnetic field dependency of *E* can be utilized to detune electromechanical resonance for magnetic field sensing. Gojdka et al. [38] reported the effect of delta *E* on the FeCOSiB layer in an Si cantilever for the first time.

**Figure 7:**
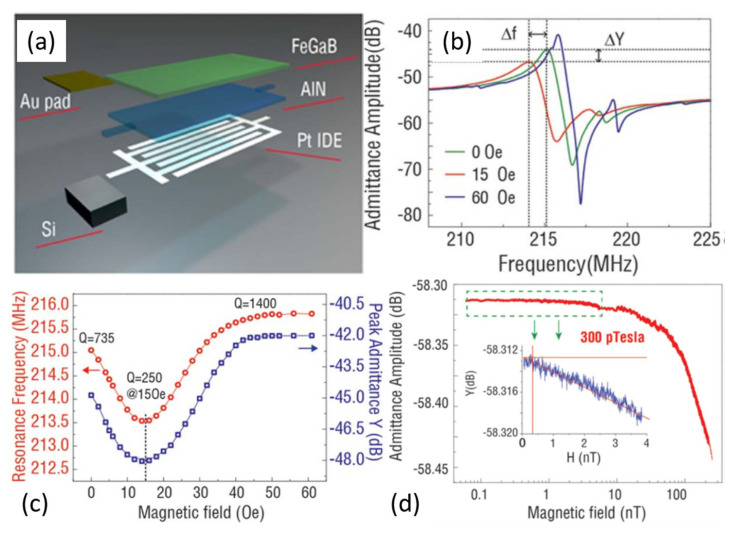
(**a**) Schematic illustration of the NEMS sensor. (**b**) Admittance curve of the NEMS sensor at various *H*_dc_. (**c**) *H*_dc_ dependence pf the *f*_r_ and admittance amplitude. (**d**) The sensitivity of the magnetic field sensor. The inset shows the zoomed in part from 50 pT to 4 nT. Reprinted with permission from ref. [39]. Copyright 2021 Springer Nature Publishing.

Nan et al. [39] demonstrated an FeCoSiB/AlN ME sensor based on the delta-E effect at *f*_r_ of 215 MHz. The structure of FeCoSiB/AlN ME sensor is shown in the Figure 7a. The AlN/(FeGaB/Al_2_O_3_) × 10 magnetoelectric heterostructure consists of AlN layer with seven Pt inter-digital electrodes on the bottom side and highly piezomagnetic low-loss RF (FeGaB/Al_2_O_3_) × 10 multilayer as the electrically floating top electrode. Figure 7b shows the admittance curve of the FeCoSiB/AlN ME sensor at various DC bias magnetic fields. They observed a shift in frequency under an external magnetic field owing to the change in Young’s modulus. Both the resonance frequency and admittance amplitude initially decrease with increasing *H*_dc_, reaching minimum at *H*_dc_ of 15 Oe, and then increased until saturating at 10 Oe to 60 Oe (Figure 7c). To determine the ME sensitivity, they recorded the magnitude of peak admittance with a decrease of the magnetic field from 200 nT to 0.1 nT, as shown in Figure 7d. The admittance amplitude starts to scatter at 300 pT, indicating the minimum detectable DC magnetic fields of 300 pT. Similarly, they achieved the minimum detectable field of 600 pT under self-biased conditions [39]. Jahns et al. [33] reported a sensitivity of 100 Hz/mT with an LOD of 12 nT/ Hz^1/2^ at 10 Hz using the frequency shift method in a delta-E effect ME sensor. Similarly, Ngoc [38] reported a DC magnetic field detection using frequency shift data for PZT/Tb–Fe–Co ME thin films. An AC electric field was used in place of a magnetic field for the frequency shift. They obtained an ME sensitivity of 249 Hz/T at a high field of >0.2 T and 487 Hz/T at a low field of <0.2 T. Li et al. [40] reported an ME sensitivity of 2.8 Hz/nT and LOD of 800 pT/ Hz^1/2^ for an AlN/FeGaB ME sensor. 

**Figure 8:**
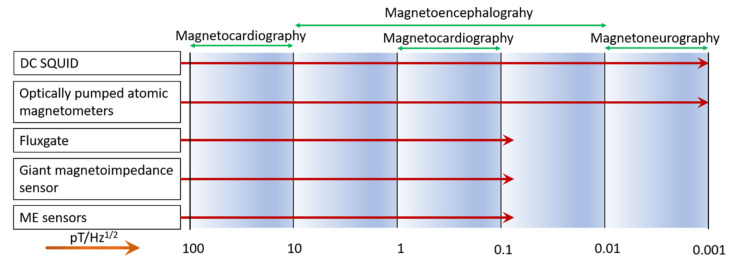
Current available various magnetic field sensors for the detection of biomagnetic signals. Reproduced from ref. [41].

Different sensors capable of detecting the corresponding biomagnetic signals are shown in Figure 8. SQUID sensors and optically pumped atomic magnetometers are well-established techniques for biomagnetic signal detection. SQUID is the most reliable technique, with an LOD of a few fT/Hz^1/2^. However, SQUID needs to be operated at liquid helium temperature and requires noise optimization and miniaturization. In contrast, optically pumped atomic magnetometers and giant magnetoimpedance sensor techniques work at room temperature but are rather complex. In comparison ME sensors with noise levels of 5.1 pT/Hz^1/2^ at 1 Hz are promising for the detection of biomagnetic signals in unshielded environment. 

## 3. ME Antennas 

**Figure 9:**
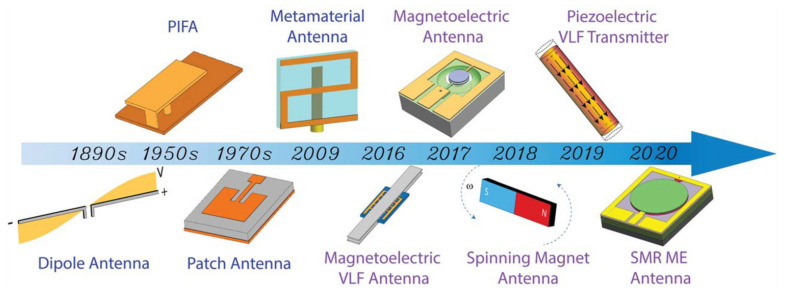
Development of compact antennas from the dipole antenna to the mechanical antenna and ME antenna. Reprinted with permission from ref. [42]. Copyright 2021 AIP Publishing.

ME antennas that convert AC current into electromagnetic (EM) waves and EM waves into AC currents have been widely studied for application in implantable medical devices, radio frequency (RF) identification systems, smart phones, tablets, radars, etc. However, conventional mechanical antennas rely on EM wave resonance; therefore, their size exceeds *λ*_0_/10, (*λ*_0_—EM wavelength), which limits their application to device miniaturization. The size limitation of the antenna makes it difficult to achieve compactness, especially at very high frequency (VHF, 30–300 MHz) and ultrahigh frequency (UHF, 0.3–3 GHz) with a large *λ*_0_. A brief history of the development of antennas from conventional to compact mechanical types is shown in Figure 9 [42]. Antennas based on the concept of ME heterostructures have been recently introduced [43,44,45]. ME antennas utilize the direct ME effect and converse ME effect in ME heterostructures. ME antennas exploit the piezoelectric and magnetostrictive properties of ME heterostructure constituents to receive and transmit electromagnetic waves [46].

**Figure 10:**
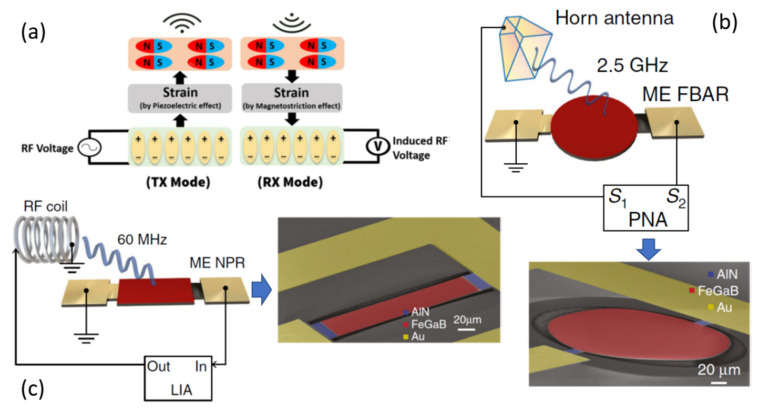
(**a**) Schematic conceptualizing transmitter (*T*_x_) and receiver (*R*_x_) modes in ME antenna. (**b**) Schematic of the ME thin-film FBAR with the antenna measurement setup where horn antenna and ME FBAR are connected to the network analyzer consisting of *S*_1_ and *S*_2_ ports. The bottom figure shows the SEM image of the ME FBAR. (**c**) Schematic of the ME NPR and the ME measurement setup using a high-frequency lock-in amplifier (HFLIA). The side figure shows the SEM image of the ME NPR. Reprinted with permission from ref. [46,47]. Copyright 2021 Springer Nature Publishing.

Figure 10a presents the concept of operating modes in the ME antenna composed of magnetostrictive and piezoelectric layers, respectively [46]. In the transmitter (*T*_X_) mode, an RF voltage is applied to the piezoelectric layer, which generates a mechanical strain in the piezoelectric layer. The generated strain is then mechanically transferred to the magnetostrictive layer through interfacial coupling, which subsequently radiates the EM wave owing to a piezomagnetic effect. In receiver (*R*x) mode, the incoming EM wave towards the magnetic layer generates a magnetostrictive strain, which subsequently transfers to the piezoelectric layer, resulting in an output RF voltage. 

**Figure 11:**
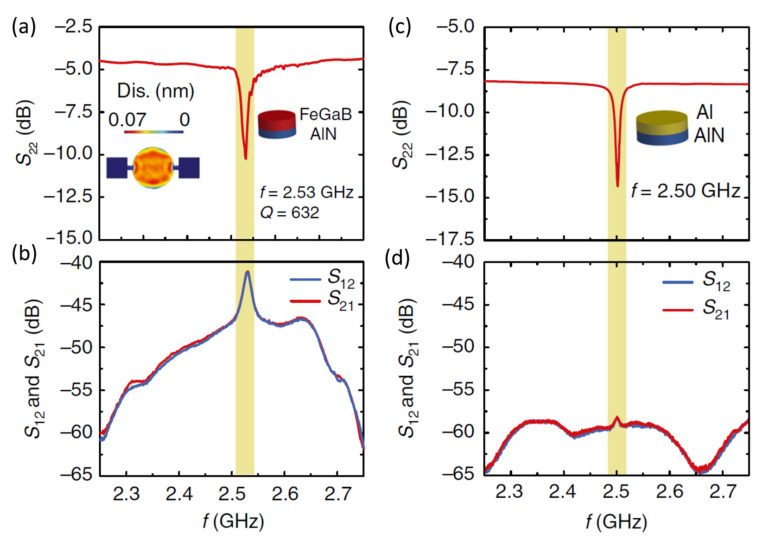
(**a**) Return loss curve (*S*_22_) of ME FBAR. The inset shows the out-of-plane displacement of the circular disk at *f*_r_. (**b**) *S*_12_ and *S*_21_ of the ME FBAR. (**c**) *S*_22_ curve of the nonmagnetic Al/AlN control FBAR. (**d**) *S*_12_ and *S*_21_ of the nonmagnetic Al/AlN control FBAR. Reprinted with permission from ref. [47]. Copyright 2021 Springer Nature Publishing.

Based on this concept, Sun et al. [47] recently demonstrated ME nanoelectromechanical system (NEMS) antennas based on two different structures: a nanoplate resonator (NPR) and a thin film bulk acoustic resonator (FBAR) operating at both VHF and UHF, shown in Figure 10b,c, respectively. These structures operate at their acoustic resonance instead of EM resonance, resulting in antenna sizes that are smaller in orders of magnitude than those of conventional antennas. These two different structures were designed for highly efficient low-frequency magnetic field sensing at MHz resonance (NPR), efficient wireless power transfer and data communication at GHz (FBAR), and simultaneous operation at MHz for magnetic field sensing and wireless communication at GHz by fabricating both the structures on the same Si wafer. The structure of the ME FBAR is shown in Figure 10b. The structure consists of a suspended FeGaB/AlN ME circular disc with a horn antenna. The FBAR structure operates at the thickness resonance frequency (*f*_r,FBAR_) of FeGaB/AlN. The *f*_r,FBAR_ of FBAR is found to be 2.53 GHz from reflection coefficient (*S*_22_) measurement as shown in Figure 11a, which also exhibits peak return loss of 10.26 dB and a *Q* of 632. Figure 11b shows the transmitting and receiving behavior of ME antennas corresponds to *S*_12_ and *S*_21_, respectively. *S*_12_ and *S*_21_ nearly overlap with each other. The calculated gain from these parameters was −18 dBi at *f*_r,FBAR_. To confirm the ME contribution, a nonmagnetic Al/AlN device was also tested using the same setup. As shown in Figure 11c,d, Al/AlN device exhibits similar electromechanical properties as FeGaB/AlN. The much lower gain for the Al/AlN structure (Figure 11d) suggests that ME coupling is dominant in the radiation of the FBAR antennas. They also fabricated NPR and FBAR structures on a single Si chip using the same fabrication process. The *f*_r,NPR_ is operated in the width (*W*) resonance mode covering a wide range of frequency bands from 60 MHz to 1500 MHz, whereas *f*_r,FBAR_ is operated in the thickness (*t*) resonance mode of 2500 MHz.

**Figure 12:**
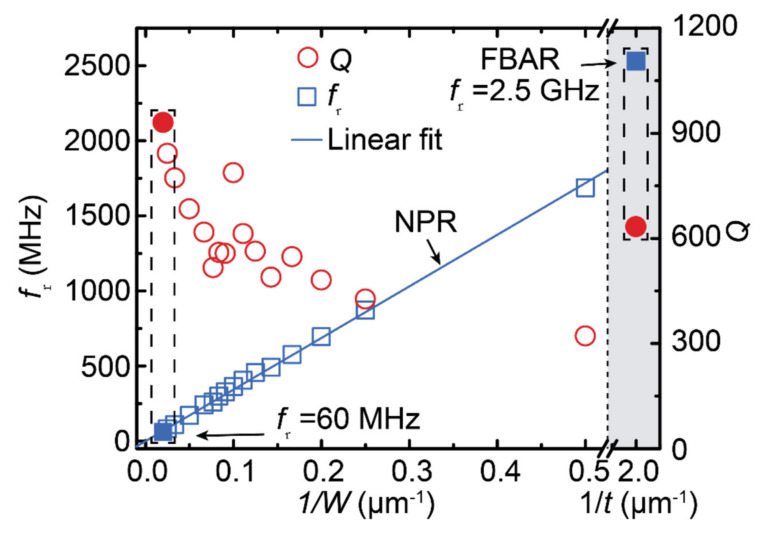
Measured resonance frequency, *f*_r_, and Q-factor as a function of 1/*w* (*w* is width) for NPR devices and 1/*t* (*t* is thickness) for FBAR devices. By using a simple device geometry design, we can achieve a wide frequency band from tens of MHz to tens of GHz on only one chip. Reprinted with permission from ref. [47]. Copyright 2021 Springer Nature Publishing.

By optimizing the device geometry through simulation, a broad band ranging from tens of MHz to tens of GHz was achieved as shown in Figure 12. A bank of multifrequency MEMS resonators can be connected to a CMOS oscillator circuit to realize a reconfigurable ME antenna array. Sun et al. [47] also reported an ultracompact wireless dual-band NEMS antenna for implantable medical devices. The antenna exhibited two acoustic resonance frequencies at 63.6 MHz and 2.51 GHz, operated for the low-frequency magnetic sensing and wireless RF energy harvesting, respectively. The wireless power transfer efficiency of the antenna was found to be 1–2 orders higher than that of other reported miniaturized microcoil devices. The magnetic field detectivity in the range of 300–500 pT allows it to record neural magnetic fields. Liang et al. [48] introduced solidly mounted resonator (SMR) antennas to further enhance the mechanical stability and high gain performance of freely suspended FBAR antennas. They fabricated an FBAR structure on top of a Bragg acoustic reflector composed of alternating layers of low acoustic and high acoustic impedance thin film layers. The Bragg reflector helps to confine the acoustic energy within the resonator, which results in enhanced radiation efficiency and higher gain [48].

## 4. ME Tunable RF/Microwave Inductors

**Figure 13:**
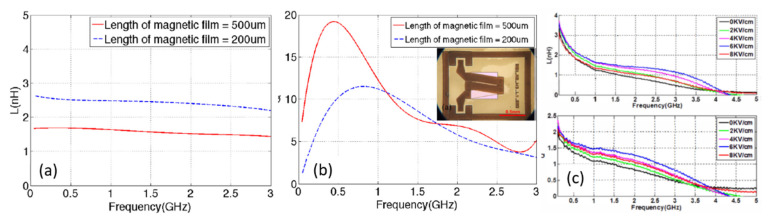
Frequency dependence of (**a**) inductance and (**b**) quality factor of FeGaB/Al_2_O_3_ multilayer thin films. (**c**) High tunability of inductance and quality factor under applied electric field for the FeGaB/Al_2_O_3_ inductor. The inset of Figure 10b shows the optical image of the inductor. Reprinted with permission from ref. [52]. Copyright 2021 IEEE Publishing.

Inductors, one of the key components of electronic circuits, are widely used in different RF applications. However, many conventional voltage-tunable inductors exhibit disadvantages such as low inductance and quality factors at the RFs [49]. One solution is to use magnetic-material-derived inductors. Salvia et al. [50] reported a permalloy-film-based tunable inductor. The use of permalloy resulted in a 40% increase in inductance, 15% tuning range, and *Q* up to 11 at 5 GHz. However, the inductor consumes considerable power to generate the required magnetic bias field. This drawback was overcome by Lou et al. [51] by using a solenoid inductor with an ME heterostructure core that exhibited an inductance of approximately 450%, with less power consumption. Later, Gao et al. [52] demonstrated an RF-integrated magnetic inductor based on a solenoid using FeGaB/Al_2_O_3_ multilayer thin films. Figure 13a,b show the frequency dependences of the inductance and quality factor for two different gaps between the neighboring windings. The inductor exhibited a significant enhancement in inductance, with a flat response over a large frequency range between 0.5 and 3 GHz as shown in Figure 13a. Similarly, as shown in Figure 13b, the inductor exhibited a high *Q* of 19 at 0.5 GHz, which can be attributed to the reduction of eddy current loss, high permeability, and lower out-of-plane anisotropy. 

To further enhance the performance of the inductor, the ME heterostructure was formed by directly bonding the tunable inductor to the PMN-PT slab [52]. Figure 13c shows the measured inductance and quality factor at different electric fields in the range of 0 to 8 kV/cm. The strain-mediated ME coupling resulted in an enhanced inductance of up to 150% and a quality factor of more than two over the large frequency range of 2–3.5 GHz, which indicates great promise as a voltage-tunable RF integrated circuit. 

## 5. ME Tunable RF/Microwave Filters

Band-pass filters are devices that allow signals between two specific frequencies to pass and block signals at other frequencies. These filters are essential to wideband communication and e-radar systems. Conventional RF/microwave filters are based on yttrium iron garnet resonators [53]. However, these filters are bulky, hindering the miniaturization of the devices. An alternative to filter miniaturization is the use of ME heterostructures that are compact, power efficient, and cost effective. The first integrated nonreciprocal dual E-field and H-field tunable band-pass filter was reported by Lin et al. [54]. They fabricated a NiZn ferrite slab on a PMN-PT slab poled along its length. The central frequency of BPF was tuned from 3.78 to 5.27 GHz with an insertion loss of 1.73–3.42 dB and an isolation of more than 13 dB under the applied magnetic fields from 100 to 400 Oe. Similarly, under an applied electric field of 0 kV/cm to 4 kV/cm, the BPF showed a frequency shift from 2.075 to 2.295. 

**Figure 14:**
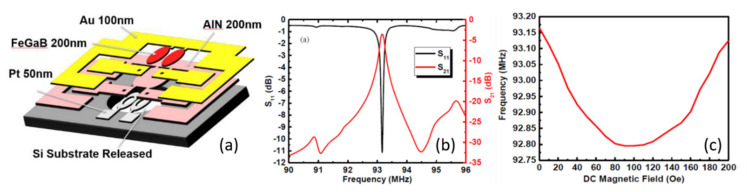
(**a**) Schematic of the layered structure of the NEMS ME band‒pass filter. (**b**) Measured return loss *S*_11_ and insertion loss *S*_21_ at zero bias field. (**c**) Resonance frequency of as a function of DC magnetic fields. Reprinted with permission from ref. [55]. Copyright 2021 IEEE Publishing.

Recently, dual E- field-tunable and H-field-tunable high Q miniaturized RF band-pass filters based on NEMS were reported by Lin et al. [55] The structure of NEMS is shown in Figure 14a. The NEMS was fabricated using two elliptical resonators, using FeGaB/Al_2_O_3_ film as magnetostrictive layers and AlN as piezoelectric layers. The two resonators are in close proximity with each other, with a separation of 2 µm. The working principle of the resonator is based on the NPR structure, as discussed in the previous section. The measured return loss *S*_11_ (the loss of power in the reflected signal) and insertion loss *S*_21_ (the loss of power resulting from the insertion) of the filter at zero bias field is shown in Figure 14b. The *S*_11_ of 11.15 dB and *S*_21_ of 3.57 dB with a high *Q* of 252 were obtained at 93.165 MHz. The band-pass filter showed a magnetic-field-dependent operation frequency. A magnetic field tunability of ~0.5% (~50 kHz/10 Oe) was observed, as shown in Figure 14c. The observed resonance frequency tunability due to ME coupling is smaller than that due to the delta E effect. This can be attributed to the remarkable change in the Young’s modulus (up to 70%), owing to the delta-E effect found in the FeGaB film. Most importantly, NEMS-based tunable RF band-pass filters are compact and power-efficient. 

## 6. ME Thin Film Energy Harvesters

Magneto-mechano-electric (MME) generators based on direct ME effect in ME composites have gained significant attention for the development of self-powered IoT devices because these generators exhibit the potential to continuously scavenge electricity from stray magnetic fields (<1 mT at 50/60 Hz) as well as vibrations. These generators comprise a resonating cantilever structure with high-performance piezoelectric materials fabricated on highly magnetostrictive substrates (Ni, metglas, Galfenol, etc.). More recently, significant improvements in the performance of MME harvesters have been achieved by exploiting their intrinsic parameters, such as adopting high-performance piezoelectric and magnetostrictive materials, modulating the geometric structures, distributing magnetic torque from magnetic mass, and using magnetic flux concentrators [56,57,58,59,60,61,62,63,64,65,66,67]. The power outputs in the range of microwatts to milliwatts were obtained using these MME generators to successfully operate the autonomous Internet-of-Things (IoT) sensors. However, these devices are prone to weak ME coupling because of unreliable bonding between the piezoelectric and magnetostrictive layers, low quality factor (Q), and high eddy current losses. Most importantly, these generators are very bulky and cannot be miniaturized. 

**Figure 15:**
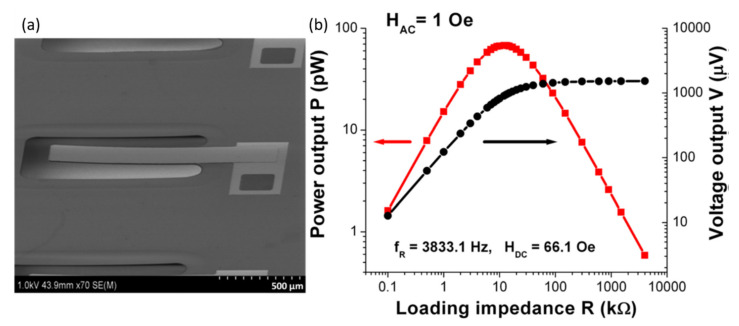
(**a**) SEM image of the all-thin-film ME energy harvester. (**b**) Energy harvesting performance of the ME energy harvester at the resonance frequency of 3833.1 Hz at *H*_dc_ = 66.1 Oe. The harvested AC magnetic field is 1 Oe. Reprinted with permission from ref. [69]. Copyright 2021 AIP Publishing.

There are very few reports available on miniaturized ME energy harvesters based on the ME composite thin films. Onuta et al. [68] developed all-thin-film ME energy harvesters on Si micromachined cantilevers. The structure of the energy harvester is shown in Figure 15a. The harvester is composed of freestanding PZT/FeCO cantilevers fabricated on an Si substrate with a plasma-enhanced chemical vapor deposited silicon oxide/nitride/oxide stack. A PZT layer (500 nm) and FeGa layer (500 nm) were spun and sputtered at room temperature, respectively. A four-mask photolithographic process was used to fabricate the 950 lm long and 200 lm wide free-standing cantilevers. Figure 15b shows the energy-harvesting performance of the single all-thin-film energy harvester. The output performance of the device was measured at a resonance frequency of 3833 Hz at an applied DC magnetic field of 66.1 Oe. A maximum power density of 0.7 mW/cm^3^ was observed at a load resistance of 12.5 kΩ. Gupta et al. have reported the PZT/Ni thin films for possible application as energy harvesters. They deposited PZT think film on Ni foil using PLD technique. They studied effect of different PLD process parameters the ME coupling of PZT/Ni thin films. They found the ME voltage coefficient of 94.5 V/cm·Oe, which is large enough for harvesting electromagnetic energy for suitable applications. Nguyen et al. [69] developed AlN/Ni, AlN/Fe, and AlN/Co thin-film ME composites and studied their energy-harvesting performance.

**Figure 16:**
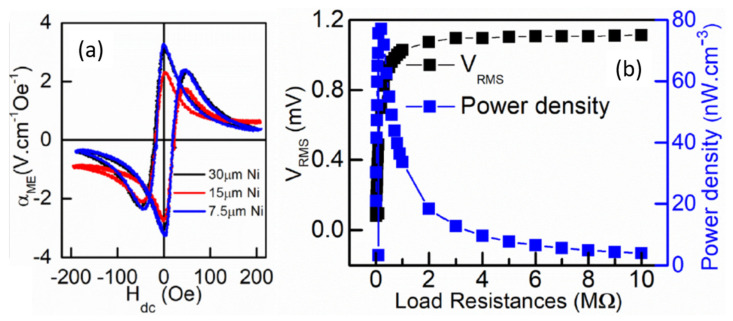
(**a**) ME voltage coefficient as a function of *H*_dc_ at *H*ac = 8.5 Oe, for different Ni foil thickness. (**b**) Off resonance energy harvesting performance of the ME energy harvester. Reprinted with permission from ref. [70]. Copyright 2021 ACS Publishing.

A strong self-biased ME voltage coefficient of 3.3 V/cm·Oe for the AlN/Ni thin film composite at an off-resonance frequency of 46 Hz was achieved as shown in Figure 16a. Figure 16b shows the measured output voltage and output power as a function of load resistance for AlN/Ni ME thin film. They obtained a maximum power density of 75 nW/cm^3^ at a load resistance of 200 kΩ.

Recently, Zaeimbashi et al. [46] reported a dual-band energy harvester and ME sensor based on VHF and UHF ME antennas, as mentioned in the previous section. The energy-harvesting mechanism is based on the *R*_x_ mode, as shown in Figure 10a. They used an FBAR structure which works in the thickness resonance mode of the ME antenna with an *f*_r_ of 2.51 GHZ for the energy harvesting. They found that the wireless power transfer efficiency (PTE) of the ME antenna is one to two orders of magnitude higher than that of other reported miniaturized microcoils. The improved PTE allows the implantable device to operate at a higher depth inside the body, allowing wireless implantable medical devices (IMDs) to be compliant with the SAR limit. Chen et al. [70] developed an ultracompact ME antenna for wireless IMDs. They obtained an antenna gain of −54.81 dBi at *f*_r_ of 371.125 MHz with a Q-factor of 123.1. Because of the short wavelength of the acoustic wave, the antenna size is effectively scaled down (8 × 350 µm^2^), resulting in the miniaturization of the entire implantable device (730 × 940 µm^2^). 

Apart from the above-mentioned ME devices, ME composite thin films have also been utilized for the application of low energy consumption spintronics such as ME memories (ME-RAM) and memristors. Most of the above-mentioned ME thin film devices are based on the direct ME effect mechanism. The ME memories are based on the converse ME effect where the magnetization is controlled by applying electric field. With the voltage-induced magnetic states, low energy-consuming non-volatile magnetic memories and magnetic nanologics can be foreseen. The detailed work on the ME heterostructures for the spintronics has been summarized in several comprehensive review articles [71,72,73].

## 7. Conclusions 

This article reviews recent progress in the field of ME thin-film devices based on ME composites. Table 1 provide the summary of characteristic and performance of some of the ME thin film devices. 

The interface strain coupling between the ferroelectric and ferromagnetic constituents of ME composite thin films plays an important role in realizing miniaturized high-frequency ME devices. A vast progress is done on the ME magnetic field sensors in recent years. The LOD of ME sensors was greatly improved by using different techniques, such as using AC magnetic or electric fields or the delta-E effect, noise suppression by using the tuning-fork technique, and adopting exchanged biased magnetostrictive materials. The LOD in the range of a few fT/Hz^1/2^ was achieved. The high performance of ME sensors was benefited from the large piezoelectric coefficients and piezomagnetic coefficients of piezoelectric and magnetostrictive phases, respectively. However, the piezoelectric and magnetostrictive materials used to fabricated ME sensors are restricted to AlN and FeCoSiB, respectively. Therefore, there is a need to develop new piezoelectric and magnetostrictive materials such as AlScN, which has a high piezoelectric coefficient and dielectric constant. Novel NEMS ME antennas based on ME thin films were designed to operate at a very high frequency (30–300 MHz) and ultrahigh frequency (0.3–3 GHz). The receiving and transmitting characteristics of the antenna relied on the direct and converse ME effects of the thin film ME composites. ME antennas were successfully miniaturized by orders of magnitude less than that of state-of-the-art conventional antennas by optimizing the physical geometries of ME thin films to obtain longitudinal and thickness vibrations modes. The designed antennas exhibit potential application in IMDs, IoTs, and wearable antennas. However, these ME antennas still have the drawback of low gain. The optimization of ME thin films or fabrication of ME antenna arrays is needed to further improve the gain of the antennas. Furthermore, development of flexible ME ME antennas compatible with smart wearable systems is a need for healthcare, security, the Internet of Things, etc. Integrated tunable inductors and band-pass filters with dual magnetic and electric field tunability at a wide operation frequency range are also discussed. These RF devices are power-efficient and compact enough to integrated with CMOS technology. The possibility of miniaturized ME thin films for energy harvesting is also discussed. However, most of the work is carried out on bulk ME energy harvester to effectively harvest the magnetic fields and vibrations. Much less work has been done on the miniaturized thin film-based energy harvesters. The miniaturized ME antennas can be further developed as an energy harvester for wireless IMDs. In summary, the recent development in the ME composite thin films indicate their potential as multifunctional ME devices. There exists a lot of scope for the development of new materials, understanding of the new ME mechanism, scaling up of thin film growth techniques, and their integration with electronics, which will have great influence on our daily life. 

## Figures and Tables

**Table 1:** Summary of some of the ME devices and their performance.

Device	Type	ME Mechanism	Characteristics	Performance
ME sensor	1. Direct sensing (a) without self-bias (b) Tuning fork (c) self-bias 2. Frequency conversion 3. Delta-E effect	DME	Highest sensitivity at fr Noise reduction Without Hdc High ME sensitivity at low frequency via MFC or EFC Change in Young modulus with the magnetic field	LoD = 135 fT/Hz1/2 at 23.23 kHz LoD = 400 fT/Hz1/2 at 867 Hz LoD = 540 pT/Hz1/2 at 10 Hz LoD = 800 pT/Hz1/2 at 10 Hz LoD = 5.1 pT/Hz1/2 at 1 Hz
ME antenna	VHR-NPR UHF-FBAR	DME	Width vibration mode (30–300 MHz) Width vibration mode (0.3–3 GHz)	$\alpha_{E}$= 6 kV/cm·Oe Gain = −18 dBi at 2.5 GHz
Tunable RF/Microwave devices	Tunable inductors Tunable Filters	CME DME and CME	High inductance tunability High Q at GHz Nonreciprocity	Inductance tunability = 191% Q = 19 at 0.50 GHz 5 MHz/Oe and 55 MHz/(kV/cm)
Energy harvesters	Magnetic field harvesting	DME	High efficiency	P = 64.61 µW
